# Integrative effect of drought and low temperature on litchi (*Litchi chinensis* Sonn.) floral initiation revealed by dynamic genome-wide transcriptome analysis

**DOI:** 10.1038/srep32005

**Published:** 2016-08-25

**Authors:** Jiyuan Shen, Qiusheng Xiao, Haiji Qiu, Chengjie Chen, Houbin Chen

**Affiliations:** 1 Guangdong Litchi Engineering Research Center, College of Horticulture, South China Agricultural University, Guangzhou 510642, Guangdong, China

## Abstract

Floral induction in litchi is influenced by multiple environment cues including temperature and soil water condition. In the present study, we determined that a combined treatment consisting of 14-day drought imposed prior to exposure to 35-day low temperature (T3) significantly promoted litchi flowering relative to the low temperature alone (T2), suggesting an integrative effect of drought and low temperature on litchi floral initiation. Analysis of transcriptomic changes in leaves from different treatments showed that 2,198 and 4,407 unigenes were differentially expressed in response to drought and low temperature, respectively. 1,227 of these unigenes were expressed in response to both treatments, implying an interaction of drought and low temperature on expression of genes involved in litchi floral initiation. Additionally, 932 unigenes were consistently differentially expressed during floral induction between T2 and T3 plants, which potentially accounts for the difference of flowering time. Thirty-eight transcription factors out of these 932 unigenes were identified as hub genes with central roles in regulation of litchi floral induction. The expression of litchi homologs of well-known flowering genes was also investigated, and one Flowering Locus T (FT) homolog may play a crucial role in litchi flowering in responses to drought and low temperature.

Litchi (*Litchi chinensis* Sonn.) is an economically important evergreen fruit tree in southern China, and is widely distributed in the subtropics as well. The annual yield of litchi is mainly determined by successful floral initiation that is influenced by multiple endogenous and environmental cues, including temperature and soil water condition^1^,^2^. However, worldwide climate changes are likely to adversely affect floral initiation and flower development.

Low temperature plays an essential role in flowering of vernalization-dependent plants^1^, and is also required for litchi floral initiation^3^,^4^, although the cold requirement varies among the different varieties^5^. Exposure to cold temperature in winter or early spring promotes litchi floral initiation, while ambient temperature above 20 °C significantly reduces litchi flowering^6^. Unusual high temperatures in winter can result in insufficient cold accumulation for litchi floral initiation, leading to inadequate flowering. Previous studies in the field have showed that to a certain extent, drought prior to winter cold induction can promote litchi flowering by reducing the requirement of cold temperature^7^,^8^. However, drought treatment alone does not induce litchi flowering^5^. Despite the agronomic importance of this integrative regulation of litchi floral induction, little is known about the molecular basis of the interaction.

The genetic mechanism underlying the vernalization response has been intensively studied in the model plant Arabidopsis^1^,^9^,^10^. In Arabidopsis, *Flowering Locus T (FT*), encoding a florigen protein, plays a central role in promoting flowering by integrating various flowering pathways^11^. In contrast, Terminal flower 1 (TFL1), sharing a highly conserved amino acid sequence with FT, exhibits an antagonistic role in plant flowering due to a single amino acid mutation^12^,^13^. However, under non-inductive condition expression of *FT* is strongly inhibited by a MADS box transcription factor Flowering Locus C (FLC), a major floral repressor in the vernaliztion pathway, which also represses expression of another important flowering promoter *SUPPRESSOR OF OVEREXPRESSION OF CO1 (SOC1*)^14^,^15^,^16^,^17^. Prolonged exposure to low temperature (vernalization) can alleviate the repression of *FT* by decreasing expression of *FLC*, but stable repression of *FLC* requires activities of VERNALIZATION1 (VRN1)^18^,^19^. On the other hand, activation of *FT* can be facilitated by CONSTANS (CO), an important regulator in the photoperiodic flowering pathway^20^. Previous studies have shown that day length-dependent genes including *CO* can play complementary and important roles in temperature signaling in floral initiation^21^,^22^. However, the integrative effect of drought and temperature on plant flowering-genes has yet to be further studied.

In the present study, drought treatment, low temperature treatment, and drought followed low temperature treatment were imposed on potted litchi plants to investigate the effects of water stress and low temperature on floral initiation. Next generation RNA-Sequencing was applied to obtain the overview of leaf transcriptome changes occurred during floral induction. The expression profiles of unigenes from different treatments were multiply compared and characterized to identify potentially important genes involved in litchi floral initiation in responses to drought and cold. Furthermore, the co-expression networks among important genes were established according to weighted gene co-expression network analysis (WGCNA). The hub genes that potentially play central regulative roles in litchi floral induction were also isolated. Together, the results provide evidence for regulation of litchi floral induction, and establish a foundation for advanced research on functional flowering-genes in litchi or similar plants.

## Results

### Drought treatment promotes litchi flowering

**Figure 1:**
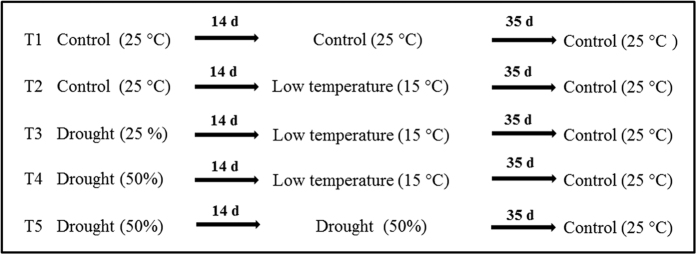
The experiment scheme of litchi floral induction conducted in a greenhouse.

**Figure 2:**
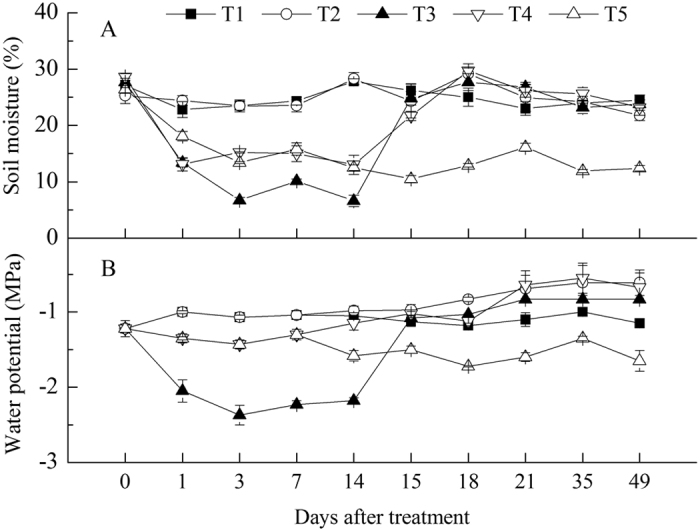
The absolute soil moisture in the pots (**A**) and water potential of litchi leaves (**B**) under different treatments.

To study the effect of drought on litchi floral initiation, potted litchi trees were subjected to treatments as indicated in Fig. 1 to permit control and monitoring of soil moisture. When the plants were fully watered, the soil moisture was about 28%, and the corresponding leaf water potential was near -1 MPa. Under the drought treatments, soil moisture levels were maintained at around 8% (T3) and 15% (T4 and T5) corresponding to about 25% and 50% of control, and the corresponding water potentials were around −2.5 MPa and −1.5 MPa, respectively (Fig. 2). The trees were fully watered when the low temperature treatment started.

After the treatments, plants were transferred to room temperature, fully watered, and the flowering time and the flower quantity were recorded. There were no flowers in T1 plants, and T5 plants rarely flowered, suggesting that low temperature is required for litchi floral initiation. All of the plants under T2, T3 and T4 treatments bloomed, even though the flower quantity, such as rates of flowering shoots, length and width of panicle of each treatment varied (Table 1). Importantly, T3 and T4 plants were found to flower about six and three days earlier, respectively, than T2 plants, indicating that certain pre-treatment of drought prior to low temperature induction promoted litchi flowering, consistent with the previous study in open field^8^. In the next year, T2 and T3 treatments were repeated to validate the promotion of floral initiation by drought. The results showed that the T3 litchi plants flowered about four days earlier in comparison with T2 plants, and the percentage of flowering plants of T2 plants decreased to 25% while 100% of T3 plants flowered.

### Analysis of dynamic changes in leaf transcriptome with RNA-Seq

To investigate the causes of differential flowering time between T2 and T3 plants, the dynamic changes in leaf transcriptome profiles were obtained by high-throughput RNA-Seq. After removing possible rRNA reads, each library produced 19.9 to 32.0 million clean reads (Table 2). These clean reads were assembled into unigenes by using the litchi genome database as a reference (http://litchidb.genomics.cn) with the mapping rate ranging from 88.7% to 92.4% for each library. Between 34,067 to 41,173 unigenes were obtained with a mean length of 1,371 bp and an average N50 length of 1,735 bp. Approximately, 91% of these unigenes had top matches to 8 species based on Nr annotation (Figure S1). More than half of these unigenes could be annotated with gene sequences from two Citrus species, i.e. *Citrus clementina* and *Citus sinensis*. In comparison with our previous RNA-Seq data from litchi leaf and bud^23^, the average length of unigenes is more than doubled, likely due to advances in the sequencing technology.

### Genes in responses to drought and low temperature during litchi floral initiation

**Figure 3:**
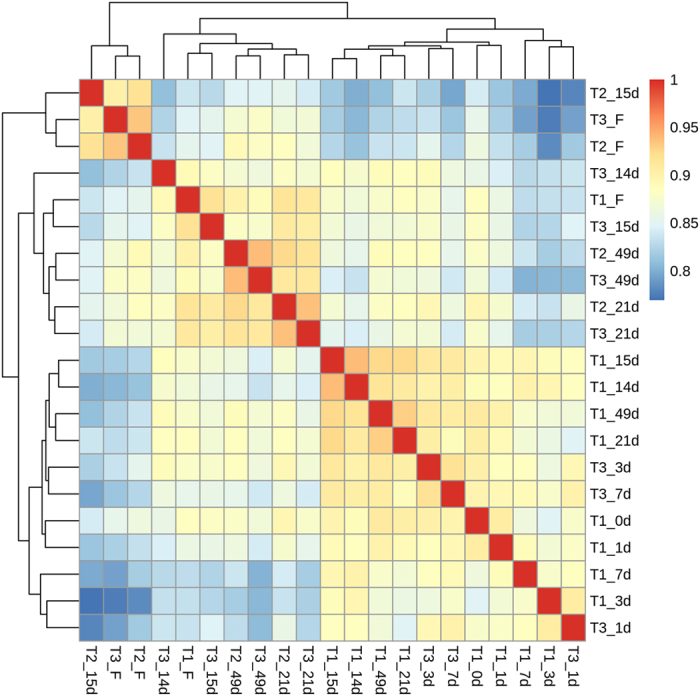
Heat map showing the spearman correlation coefficient of pairwise comparison between samples. The spearman correlation coefficient between two samples was calculated based on FPKM of each gene. The heatmap was constructed using R language with the function of heatmap.

The FPKM method was used to calculate the expression of unigenes, and all the transcriptome files of leaves from different time points for the different treatments were pairwise compared. Spearman correlation between samples was calculated based on FPKM values using the function “cor” in R package, and displayed in a heatmap (Fig. 3). Generally, these samples were categorized into three clusters: samples under room temperature and drought treatments, samples under low temperature treatment, and samples transferred to room temperature after low temperature treatment. Notably, samples under the drought treatment were grouped together except for the sample from T3-14d, implying that drought treatment caused obvious molecular changes on the 14th day. Importantly, the Spearman correlation coefficient of T2-15d and T3-15d is lower than that of T1-14d and T3-14d, which means the molecular difference between T1-14d and T3-14d plants was enlarged, although both of the T2-15d and T3-15d plants underwent one-day low temperature treatment, suggesting that an integrative effect of drought and low temperature is required for induction of certain stress-responsive genes. Hence, the genes in response to drought, low temperature, and even the combination need to be characterized.

**Figure 4:**
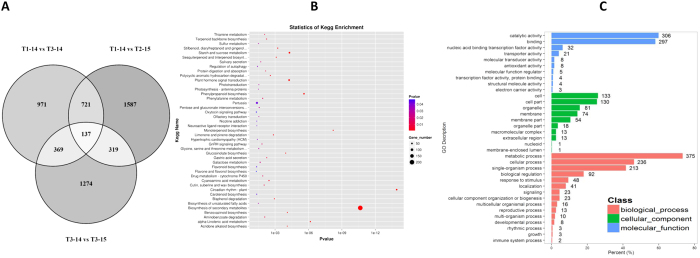
Identification and functional annotation of the differentially expressed genes (DEGs) in responses to low temperature and drought treatments. (**A**) Venn diagram of the DEGs derived from pairwise comparisons. KEGG enrichment (**B**) and Gene Ontology classification (**C**) of the genes in responses to both floral induction treatments.

According to the Venn diagram of pairwise comparisons of transcriptomes from T1-14d, T3-14d, T2-15d and T3-15d samples, 2,198 unigenes were differentially expressed in T3 plants after 14-day drought treatment (Fig. 4A). Notably, one-day low temperature treatment resulted in a different number of cold-responsible genes in T2 and T3 plants, and totally 4,407 unigenes were differentially expressed in T2 and T3 plants in response to one-day low temperature treatment (Fig. 4A), which is consistent with the earlier analysis of Spearman correlation coefficient between samples in Fig. 3. These stress-genes are potentially involved in litchi floral initiation, responding to drought and low temperature. Among these differentially expressed genes (DEGs), 1,227 unigenes were expressed in response to both drought and low temperature, suggesting an interaction of low temperature and drought on genes involving in litchi floral initiation. The KEGG analysis of these 1,227 DEGs showed that genes related to Biosynthesis of secondary metabolites, Starch and sucrose metabolism and Plant hormone signal transduction represented the major proportion (Fig. 4B), indicating the intricate network between external stress cues and endogenous metabolism and signaling. The GO analysis classified these 1,227 unigenes into three categories: biological process, cellular component and molecular function (Fig. 4C). For the biological process category, metabolic process and cellular process represented the major proportion. Under the cellular component category, large numbers of unigenes were categorized as cell and cell part. Under the molecular function category, catalytic activity and binding were the top two most abundant sub-categories.

### Genes accounting for the different flowering time between T2 and T3 litchi plants

**Figure 5:**
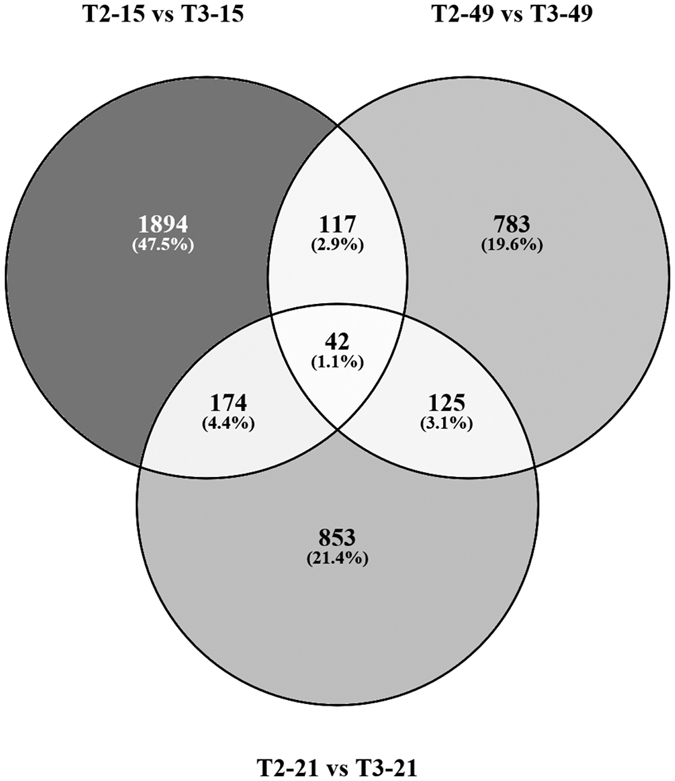
Venn diagram of the differentially expressed genes (DEGs) derived from pairwise comparisons between T2 and T3 plants at 15, 21 and 49-day time points under low temperature treatment.

T3 litchi plants flowered 3–7 days earlier than T2 plants did. To identify the gene(s) accounting for the difference, the transcriptomes of T2 and T3 plants at treatment time points of 15, 21 and 49-days were accordingly pairwise compared. Totally, there were 3,988 genes differentially expressed (Fig. 5), which might account for the difference of flowering time between T2 and T3 litchi plants. However, among these 3,988 DEGs, only 458 unigenes (11.5% of 3,988 unigenes) were differentially expressed in at least two pairwise comparisons across the low temperature treatment. Therefore, expression pattern and consistency of these potential candidate genes should be taken into account.

**Figure 6:**
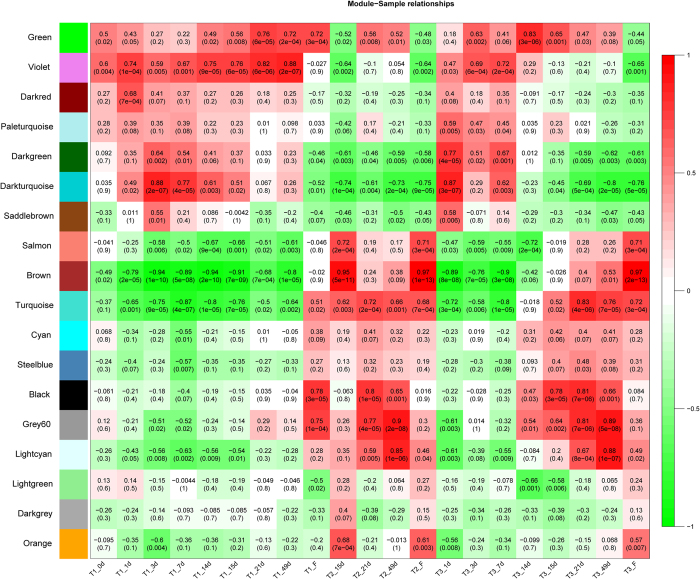
Heatmap showing the weighted gene co-expression network analysis (WGCNA) of genes between different litchi leaf samples. Each row corresponds to a module and each column corresponds to a time point of treatment. The top and bottom number in each cell indicate the correlation coefficient between the module and time point and p-value of the test, respectively.

**Figure 7:**
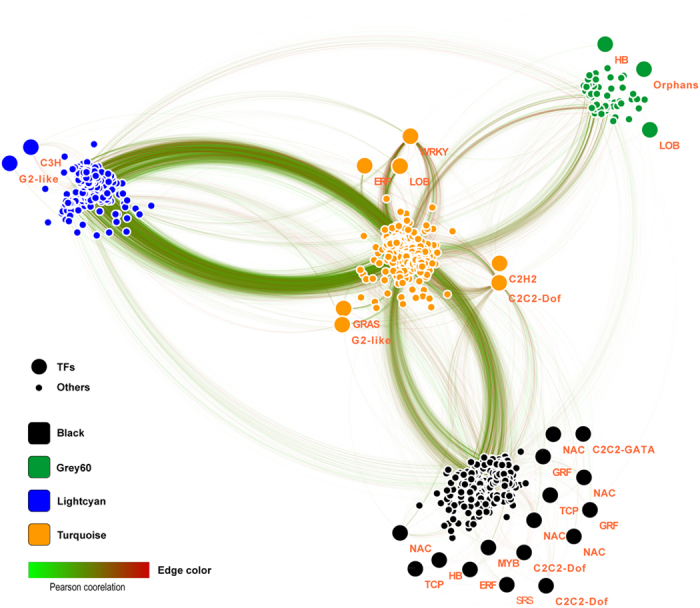
The co-expression network of the genes potentially accounting for the difference of flowering time between T2 and T3 plants. The co-expression network was constructed from 932 genes from 4 modules.

A co-expression network was established on the basis of weighted gene co-expression network analysis (WGCNA), to analyze the gene expression pattern and linkage. Totally, 24,537 unigenes that were differentially expressed in at least one pairwise comparison of samples were used for WGCNA; 24,242 unigenes were categorized into 18 distinct modules and the other 295 genes were outliers (Fig. 6). Among these modules, the bottom three show an irregular expression trend. The top seven consisted of genes mainly expressed in leaves aging under room temperature are likely to be responsible for leaf development and senescence. Of particular interest is the identification of the flowering-related genes in responses to stress. Genes expressed in responses to drought and low temperature comprise the middle eight co-expression modules, of which four (cyan, steelblue, black and grey60) display drought-response and the other four (salmon, brown, turquoise and light_cyan) show low temperature-response. However, only black, grey60, turquoise and light_cyan modules show *p*-value ≤0.05 under drought and low temperature treatments, as indicated by cluster analysis in Figure S2. The eigengene expression of these 4 modules comprised of 8,029 genes is shown in Figure S3. 932 out of the 8,029 genes are shared in the DEGs between T2 and T3 at 15, 21 and 49-day time points (Figure S4). WGCNA was carried out on these 932 genes to construct the network, in which genes are indicated as nodes and the connecting lines (edges) with red and green color correspondingly represent the positive and negative co-expression correlations (Fig. 7). The statistical connection of all these 932 unigenes in the network suggested that these genes tend to be inner-correlated. In addition, 38 out of 932 unigenes are identified as transcription factors likely playing central roles in litchi floral initiation in responses to drought and low temperature.

### Expression profiles of flowering genes in leaves during litchi floral induction

**Figure 8:**
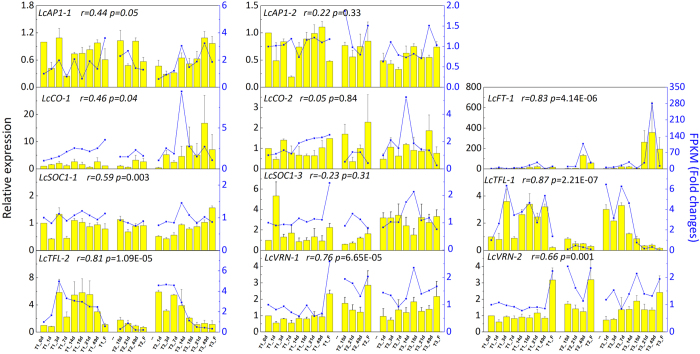
Expression confirmation of eleven flowering-related genes using real time Q-PCR. The relative expression of target genes relative to a control gene (*LcActin*) in real time Q-PCR was shown as yellow column with standard error, and their corresponding fold changes (relative to the value of T1_0d) of FPKM value from RNA-seq were shown as blue lines. The Spearman correlation coefficient (*r*) between the gene expression values derived from the two methods and associated *p* value (n = 21) were shown accordingly.

The homologues of well-known flowering-related genes in litchi were also investigated in the present study (Fig. 8 and Figure S5). As revealed by the gene expression patterns obtained from RNA-Seq data, two *CONSTANS* genes (*LcCO1* and *2*) and *LcSOC1-1* were up-regulated by drought treatment. Two *VERNALIZATION* genes (*LcVRN-1* and 2) were induced while two *TERMINAL FLOWERING LOCUS* genes (*LcTFL*-1 and 2) were reduced by low temperature exposure. Two *APETALA1 (LcAP1-1* and 2), one *FLOWERING LOCUS T (LcFT1*), and *LcSOC1-3* were induced by both treatments. Of particular importance, the induction of *LcFT1* by drought treatment was larger after exposure to low temperature resulting in significantly higher transcripts level of *LcFT1* in T3 plants compared to T2 plants (Fig. 8).

To evaluate if and how the FPKM value represents the transcript level, expression of these eleven flowering-related genes was measured using real time Q-PCR. The relative expression of eight genes was consistent with their FPKM values obtained from RNA-Seq (*r *= 0.44 to 0.87). However, expression patterns of the other three genes were not strictly correlated with the RNA-Seq data (Fig. 8), possibly due to RNA alternative splicing. Alternative RNA splicing is common, and leads to multiple transcripts (isoforms) per gene. The numbers and types of isoforms for genes are tissue-specific and vary during the life cycle^24^. Therefore, the isoforms can be accurately determined by RNA-Sequencing, but sometimes it is not easy to measure the expression by Q-PCR because it is difficult to design primers to quantify an isoform of a gene expressed in different tissue.

## Discussion

Plant flowering time is determined by endogenous signals and environmental cues, including phytohormones, photoperiod, vernalization process, and phase transition from juvenile to adult^9^,^25^,^26^. In nature, plants are usually exposed to multiple external cues and must facilitate the integration of this comprehensive information into a flowering network^21^,^27^,^28^,^29^.

In the case of litchi, low temperature is highly required for floral initiation, but unusually high temperature in winter can potentially lead to insufficient cold accumulation resulting in inadequate flowering. In an effort to enhance litchi flowering, agronomic regulation is sought to alleviate the adverse effect of climate change. In the present study conducted in a greenhouse, drought treatment followed by cold temperature induction caused earlier flowering in litchi compared to only cold temperature treatment (Table 1), which is consistent with studies in open field^8^ and other species^30^. This result is also supported by previous studies that plants tend to accelerate flowering and produce seeds in response to abiotic stress such as clod temperature, water deficit, and UV-C exposure before severe stress leads to death^1^,^31^,^32^. However, drought treatment without subsequent low temperature exposure did not induce litchi flowering^5^, and a similar result was also observed in our study, where 49-day mild drought resulted in a small number of flowers (Table 1) suggesting that drought cannot replace the low temperature to induce litchi floral initiation^33^. Although a previous study showed a dose-dependent drought effect, i.e. the effect of drought depends on the severity, duration and even the genotype^34^, intensive or long-term drought stress may negatively affect flower development and fertility^9^,^35^, reducing crop productivity.

To investigate the molecular basis of the integrative effect of drought and low temperature on litchi floral initiation, the dynamic changes in transcriptomes in leaves under different treatments were studied using RNA-Seq technology^36^,^37^. According to the overview of the transcriptomes profiles (Fig. 3), the early responses of litchi plants to drought at a molecular level are not apparent suggesting the dosage-dependent effect of drought^38^, and thus we mainly analyzed the drought-responsive genes in leaves at the 14-day time point where 2,198 genes were found to be regulated by drought. Compared to drought, one-day-low temperature treatment caused remarkable changes in gene expression and about one-third of these genes responded to drought stress (Fig. 4) implying an interaction of low temperature and drought^39^. The KEGG analysis shows that these genes responding to both low temperature and drought treatment are mainly related to biosynthesis of secondary metabolites, starch and sucrose, as well as plant hormone signal transduction, which has been suggested to be associated with floral initiation and flower development. For example, phytohormone signaling is known to participate in various plant morphogenetic processes, including the switch from vegetative to reproductive growth^40^. In Arabidopsis, GA is considered to be the major promoting signal in flowering^41^,^42^, while ABA commonly antagonizes GA signaling^40^. However, ABA likely has a positive role in floral initiation in litchi plants^43^. Sugars are traditionally regarded as metabolic resources for energy supply and carbon skeleton construction, but recently sugars have been suggested to serve as signals in the plant life cycle. Plants containing mutations in starch biosynthesis or sugars transporter genes exhibit delayed-flowering^44^,^45^. Interestingly, one-day low temperature treatment induced different sets of cold-responsible genes in control and drought-treated plants (Fig. 4) indicates the integrative effect of drought and low temperature on gene expression. This result may indicate that either drought-mediated genes are required for activation of some low temperature-response genes, or that drought-treated plants and well-watered plants respond to low temperature in quite different manners.

As a consequence of the interaction of drought and low temperature, 3,988 unigenes were differentially expressed between T2 and T3 plants (Fig. 5), 932 out of these DEGs were consistently expressed in response to drought or low temperature based on further analysis using WGCNA (Fig. 6; Figure S3). Moreover, some litchi homologs of well-known flowering genes were found in the co-expression modules derived from the 932 genes (Fig. 8). For example, one litchi *FT* homolog (*LcFT1*) from light_cyan module was induced by drought and low temperature, similar to what was observed in *Arabidopsis*^32^. Epigenetically over-expression of this *FT* homolog in *Arabidopsis* results in significantly early flowering^46^, indicating the conserved functions of these genes in litchi plants^47^,^48^.

Generally, vernalizaiton or low temperature activate expression of *FT* via reducing the expression of *FT*-repressors including FLC and TFL. In the present study, low temperature did not alter the expression of *FLC*s, but did significantly decrease the expression of *TFL*s, which potentially alleviated the repression of expression and transport of FT; thus, TFL rather than FLC seems to be the major negative flowering regulator during floral induction (Fig. 8 and Figure S5). The expression of *FT* also requires upstream activators including CO that is induced not only by long photoperiod^49^, but also by low temperature and drought, as proposed by our data (Fig. 8 and Figure S5), leading to induction of *FT* and *SOC1*^50^, and eventually promotion of flowering.

## Materials and Methods

### Plant materials

Three-year-old air layered potted litchi trees (*Litchi chinenesis* cv. Feizixiao) were grown in a green house on the campus of South China Agricultural University in Guangzhou City with loam, coconut chaff and mushroom cinder (3:1:1). The terminal growing flushes of twenty-one plants were removed one week prior to treatments for similar plant growth status for individual plant.

### Experiment scheme

Trees were randomly divided into 5 groups with 4 or 5 plants per group, and subjected to treatments as indicated in Fig. 1 in a growth chamber with adjustable temperature and humidity. In the beginning, all the plants were grown under room temperature (25 ± 2 °C); while T3, T4 and T5 plants were drought stressed with the soil moisture corresponding to 25%, 50% and 50% respectively of the T1 and T2 plants which were fully watered. For the drought treatments, the relative air humidity in the chamber was adjusted to 30% to accelerate the water evaporation. After 14 days, T2, T3 and T4 plants were transferred to a low temperature chamber (15 °C) and fully watered. However, T1 and T5 plants were kept under room temperature, with T5 plants maintained at 50% relative soil moisture. Thirty-five days later, plants were transferred to room temperature chamber and fully watered. These pots were moved daily to avoid the possible overlap of leaves. The leaf water potential and relative soil moisture were monitored when the drought treatment was carried out. The third to sixth compound leaves were sampled at 0, 1, 3, 7, 14, 15, 21 and 49-day time points named as T1-0…49d accordingly, and final sampling was occurred when any panicle primordia emerged namely T1-F. The plants for T1 and T2 treatments were not separated until low temperature treatment was imposed on T2 plants.

### Drought treatments, measurement of leaf water potential and soil moisture

Drought treatment was carried out as described by Su *et al*.^51^ with little modification. At the start, plants were fully watered and soil moisture was measured 2 h later by Theta probe type ML2x (Delta-T Devices Ltd; Cambridge England), which was indicated as soil water capacity. For the drought-treated plants, the soil water naturally evaporated until reaching the set level. Then the soil moisture was maintained by accurately watering according to the daily loss of water. Leaf water potential was measured with a pressure chamber (PMS Instrument Co., Corvallis, Oregon, USA). The soil moisture was measured three times per pot and leaf water potential was detected once per plant with the values indicated as the means ±SE of 4–5 plants.

### RNA extraction, library construction and next generation RNA-Sequencing

Total leaf RNA was extracted using Hipure Plant RNA Mini Kit (Magen, China) according to the manufacturer’s instruction and treated with DNase (Takara, China). Equal quantities of RNA from three biological replicates were pooled to construct libraries that were sequenced with Illumina HiSeq^TM^ 2500 using a 125 paired-end (PE) module at ANNOROAD Company (Beijing, China). The raw reads were trimmed and filtered using Trimmomatic software (v0.33) to remove adapters and low quality bases. The high-quality reads were aligned to the sillva SSU and LSU ribosome RNA (rRNA) database, and the matched reads were removed to generate clean reads.

### Assembly and annotation

Clean reads were assembled into transcripts using Cufflinks with the litchi genome as a reference. Cuffmerge was used to assemble the transcripts into non-redundant unigene sets. The functions of unigenes were annotated by blastXing to NCBI non-redundant (nr) database, Swiss-Prot protein database and Kyoto Encyclopedia of Genes and Genomes (KEGG) database with E-value threshold of 10^−5^ and minQueryCo (v0.33). To generate a broad overview of groups of genes, Gene Ontology (GO) classification was applied. GO annotation was obtained by id conversion from NCBI gene ID number to GO id leveraging the mapping relation downloaded from PIR (ftp://ftp.pir.georgetown.edu/databases/idmapping/idmapping.tb.gz) using TBtools software (http://www.cjchen.name/TBtools.jar).

### Gene expression profiling

All sequences of transcripts were extracted from reference genome sequence using gffread from cufflinks pipeline. The expression calculation at transcript and gene level was conducted using RSEM (v1.2.21) with default parameters. “Fold change ≥2, *p* value ≤0.05” was set as the threshold for significant difference in expression.

### Construction of gene co-expression networks and heat map

The ‘cor’ function in R programming language and software (http://www.r-project.org/) was used to calculate Spearman correlation coefficients between samples. A co-expression network was constructed using weighted gene co-expression network analysis (WGCNA, v1.48) package in R. Totally 24,537 genes that were differentially expressed in at least one pairwise comparison of samples were used for analysis. The log_2_ (FPKM + 1) values of these genes were imported into WGCNA to generate the modules with default setting except that the soft power is 5, min module size is 100, deep split is 1 and merged cut height is 0.25. The total connectivity and intra-modular connectivity (function soft Connectivity), eigengene-based connectivity (kME), and kME-P value were calculated for the 24,242 genes, which were clustered into different modules and remaining 295 genes were outliers. The heat map was constructed using the R package “pheatmap”.

### Real time quantitative PCR (Q-PCR) analysis

Relative expression of flowering genes in leaf tissues was measured using Q-PCR performed on LightCycler 480 II (Roche, Germany) with iTaq Universal SYBR Green Supermix (BioRad, USA) according to the manufacturer’s instruction. For each sample three biological replicates were taken based on which the average value and standard error were calculated. PCR amplification was conducted under the following conditions: 95 °C for 10 min, followed by 40 cycles at 95 °C for 10 sec, then at 60 °C for 20 sec and finally 72 °C for 20 sec. PCR products were melted at 95 °C for 5 sec, and then 65 °C for 1 min to detect the specificity. The gene expressions were normalized against an internal reference gene *LcActin* (HQ615689). Primers used in this study were designed on website of Primer 3 (v 0.4.0; http://bioinfo.ut.ee/primer3-0.4.0/primer3/) and showed in Figure S6.

## Additional Information

**How to cite this article**: Shen, J. *et al*. Integrative effect of drought and low temperature on litchi (*Litchi chinensis* Sonn.) floral initiation revealed by dynamic genome-wide transcriptome analysis. *Sci. Rep.* **6**, 32005; doi: 10.1038/srep32005 (2016).

## Supplementary Material

Supplementary Information

## Figures and Tables

**Table 1:** Analysis of litchi flowering time and flower quantities in responses to different induction treatments.

Treatment	Floral induction days (d)^a^	Percentage of flowering plants (%)	Percentage of flowering shoots (%)	Panicles per shoot per plant	Panicle length (cm)^b^	Panicle width (cm)^b^
T1	**/**c	**/**	**/**	**/**	**/**	**/**
T2	66.25 ± 2.59	100	75	2.05	14.45 ± 0.19	6.14 ± 1.93
T3	59 ± 0	100	100	2.49	18.61 ± 1.31	10.29 ± 1.74
T4	62.80 ± 0.58	100	81	2.67	19.98 ± 1.71	7.28 ± 0.84
T5	62 ± 0	25	20	0.20	7.93 ± 3.73	3.25 ± 1.10

^a^Floral induction days represent the period from the day of experiment started to the day panicle primordium emerged.

^b^The length and width of panicle were measured on the longest and widest parts of the biggest panicle in each inflorescence. Data values represent mean ±SE (*n *= 4 or 5).

^c^ “/” means not applicable.

**Table 2:** Throughput and quality of RNA-Seq of litchi leaves from different time points of different floral induction treatments.

	No. of Clean reads (million)	No. of unigenes	N50 (nt)^a^	Mean length (nt)	GC Percentage	Mapping rate
T1_0d	25.6	40,099	1780	1408	41.59%	90.1%
T1_1d	24.4	38,009	1754	1396	41.85%	91.4%
T1_3d	22.6	37,863	1731	1373	41.89%	91.7%
T1_7d	23.0	35,914	1630	1291	42.38%	90.9%
T1_14d	27.4	38,251	1713	1341	41.78%	91.8%
T1_15d	32.0	36,961	1768	1385	41.53%	91.8%
T1_21d	26.9	36,824	1742	1373	41.70%	91.3%
T1_49d	27.7	35,326	1755	1387	41.68%	90.9%
T1_F	22.7	36,263	1662	1304	41.73%	90.7%
T2_15d	27.2	39,731	1674	1319	41.90%	88.7%
T2_21d	24.6	38,573	1737	1367	41.60%	90.7%
T2_49d	24.0	41,173	1759	1390	41.53%	91.6%
T2_F	25.2	39,101	1735	1372	41.88%	90.5%
T3_1d	23.0	39,005	1795	1428	41.85%	89.7%
T3_3d	23.2	38,750	1685	1321	41.88%	90.3%
T3_7d	19.9	34,067	1682	1343	41.96%	91.9%
T3_14d	22.8	40,036	1708	1331	41.83%	90.7%
T3_15d	20.6	38,334	1807	1452	41.60%	92.4%
T3_21d	26.2	37,390	1821	1429	41.45%	91.6%
T3_49d	22.2	38,608	1739	1379	41.58%	91.4%
T3_F	22.4	40,033	1766	1399	41.64%	91.5%
Average	24.5	38,110	1735	1371	41.75%	91.0%
