# Supplementary material for: Integrative effect of drought and low temperature on litchi (Litchi chinensis Sonn.) floral initiation revealed by dynamic genome-wide transcriptome analysis
Source: Sci Rep. 2016 Aug 25;6:32005. doi: 10.1038/srep32005 (PMC4997319; doi:10.1038/srep32005)
Supplement: Supplementary Information [file srep32005-s1.pdf]

Integrative effect of drought and low temperature on litchi (*Litchi chinensis* Sonn.) floral initiation revealed by dynamic genome-wide transcriptome analysis

Jiyuan Shen,

Qiusheng Xiao,

Haiji Qiu,

Chengjie Cheng,

Houbin Chen*

Figure S1. Number and percentage of Unigenes matching the 9 top species using BLASTx to *nr* databse.

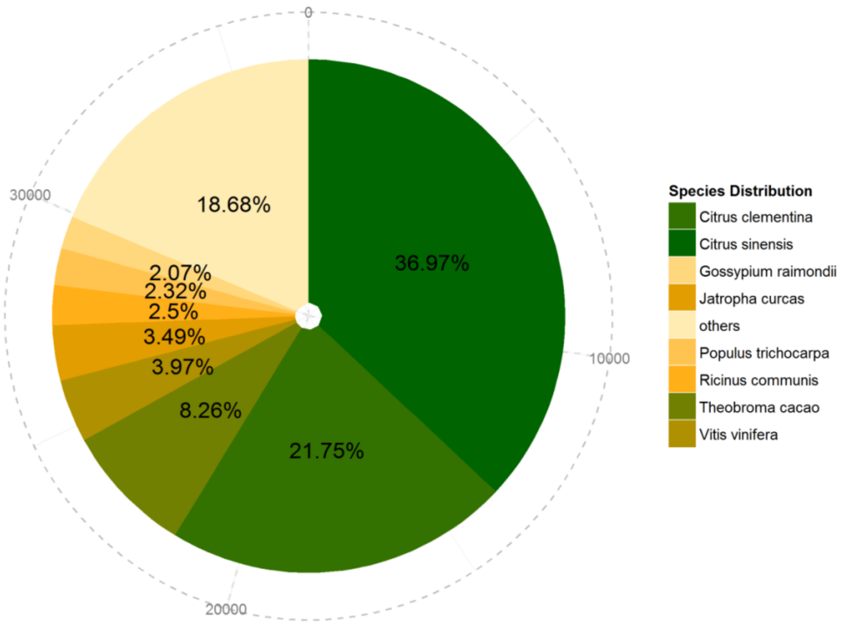

Figure S2. Cluster of co-expression modules identified by WGCNA.

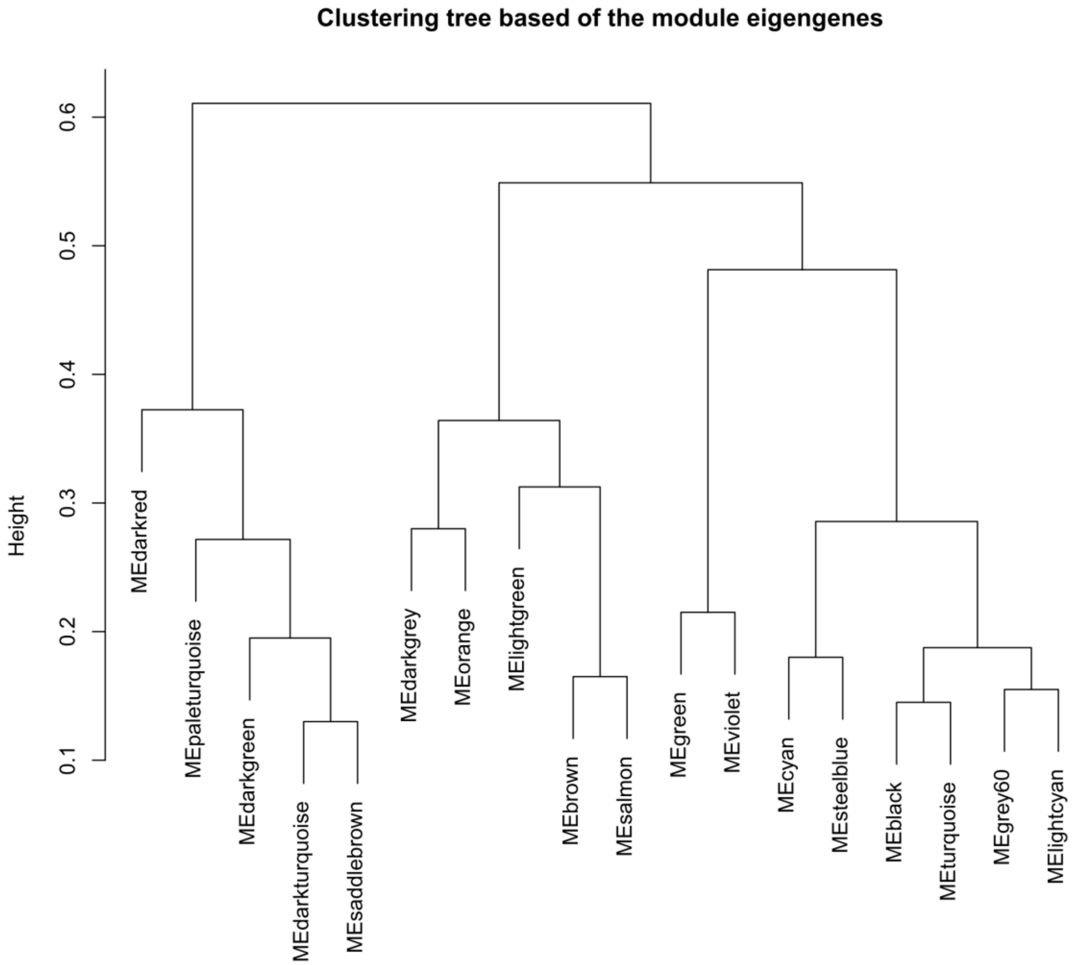

Figure S3. Eigengene expression profiles for lightcyan, turquoise, black and grey60 modules under different treatments. The y axis represents the value of the module eigengene, and the x axis represents time point of treatment.

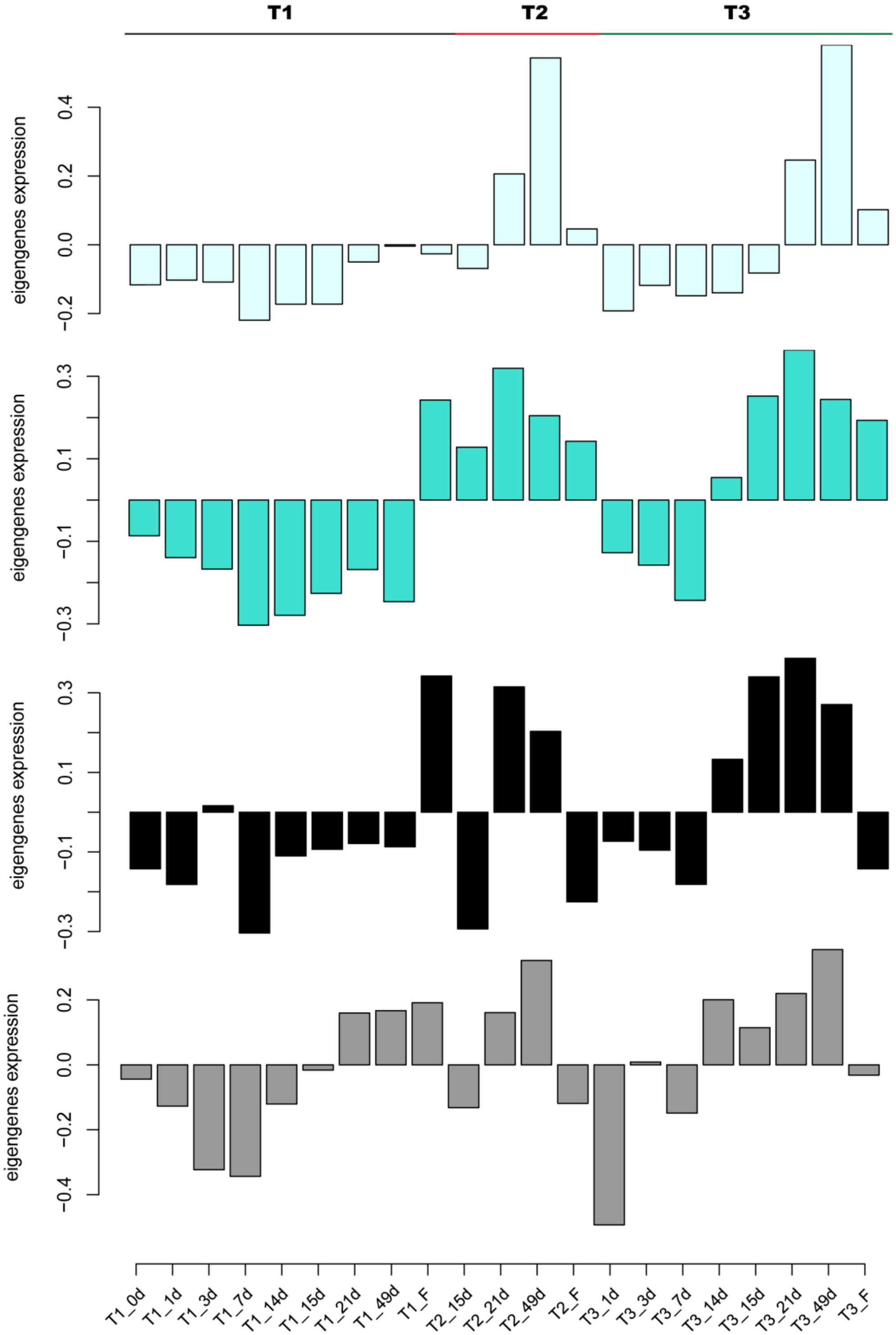

Figure S4. Venn diagram of DEGs between T2 and T3 plants at 15, 21 and 49-day (A), and genes clustered into lightcyan, turquoise, black and grey60 modules (B).

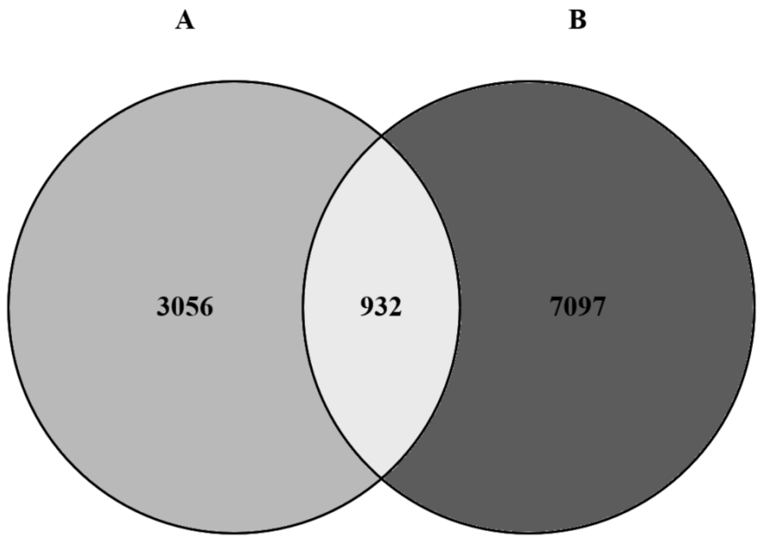

Figure S5. Expression profiles of litchi homologs of well-known flowering genes under different treatments. The FPKM values of each gene were normalized into the range of 0 to 1.

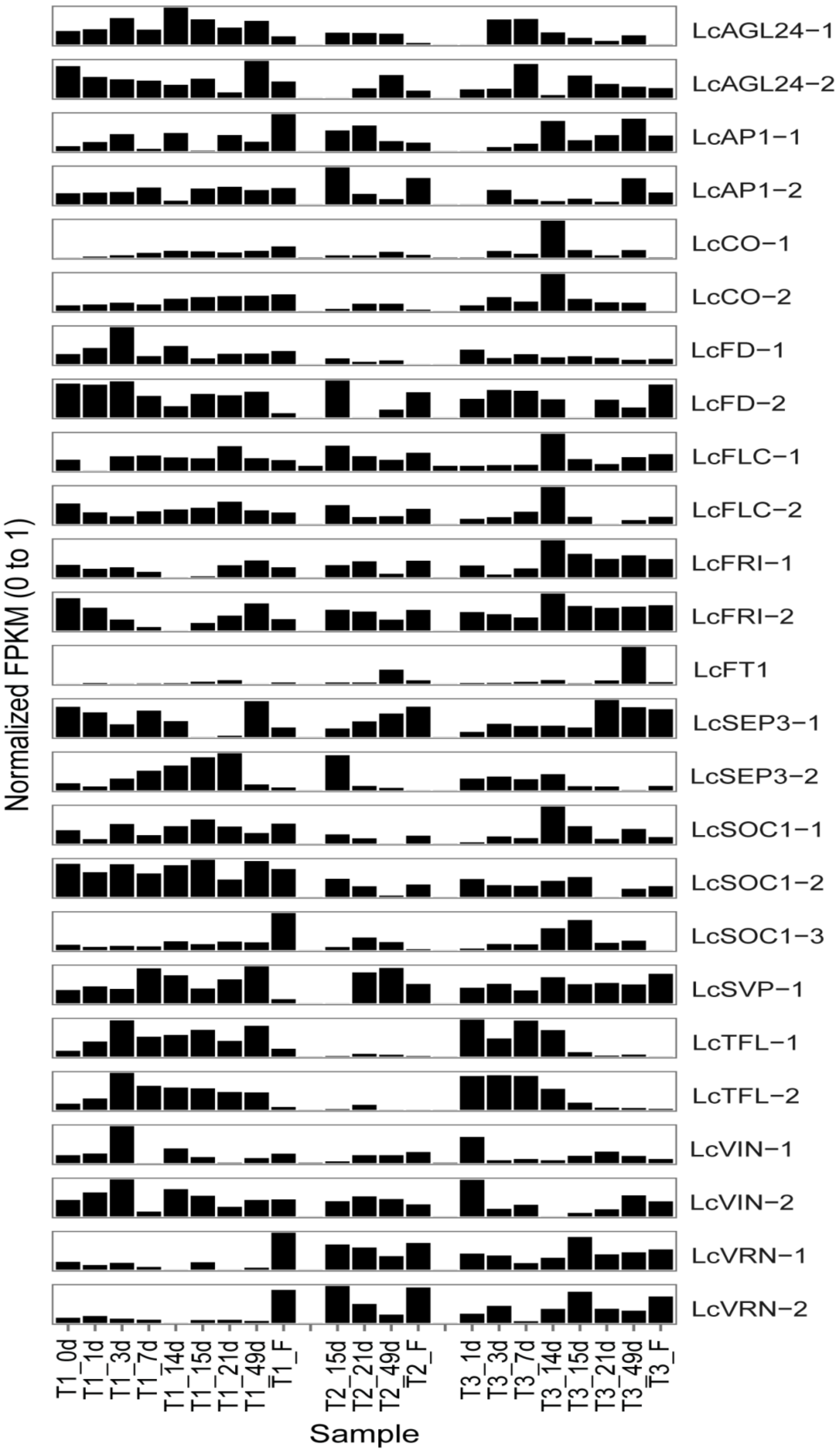

Figure S6. Primers for real time Q-PCR analysis

Gene	Forward Primer (5’-3’)	Reverse Primer (5’-3’)
LcActin	GAGTCTGGTCCATCCATTGTC	CCTCAACATGTCTCCCACATC
LcAP1-1	TCGAGCATATCAAGAGCAGTT	GGCATTCCAGAGACCAGTTT
LcAP1-2	ACAAACGCACTAGGCAAACC	GCCTTCTTTCCACCATCAGA
LcCO-1	TCCAAATTCCTAGCATGTACCA	ATCATGGGCCCACTTTCATA
LcCO-2	CAGCTCAGCATCCAAATTTCT	ATTTGGGACCCATGAGGTTT
LcFT-1	GCCGCGGTCTACTTTAATAGC	TCTTTGCTAGCTTTGACATGC
LcSOC1-1	CCTAGTAATTGCTTTTCTCTGCAAC	CCCAGATAGAGAAAGAAGAGACCA
LcSOC1-3	GTTCATAGGGCCACCTGAGA	GCAAAATAAAGCCAGCCTGT
LcTFL-1	CTAGACCCTCTGGTCGTTGG	GCCATTAGCGACCTGTTTGT
LcTFL-2	ACTGTCATCATCGTCCATCG	CAGTGAACTCCGTGACCTGA
LcVRN-1	TGCTTAGATCGAGGGAATTTG	TTGAAGAGGTTTTAGCGACCA
LcVRN-2	GGTGCAGGATTCATGAACAG	TCGGTAACAGATCACCTGAAAA
